# Outcomes of neoadjuvant and adjuvant chemotherapy in stage 2 and 3 non-small cell lung cancer: an analysis of the National Cancer Database

**DOI:** 10.18632/oncotarget.25327

**Published:** 2018-05-11

**Authors:** Matthew MacLean, Xin Luo, Shidan Wang, Kemp Kernstine, David E. Gerber, Yang Xie

**Affiliations:** ^1^ Quantitative Biomedical Research Center, Department of Clinical Sciences, University of Texas, Southwestern Medical Center, Dallas, Texas, USA; ^2^ Department of Bioinformatics, University of Texas Southwestern Medical Center, Dallas, Texas, USA; ^3^ Department of Cardio Thoracic Surgery, University of Texas Southwestern Medical Center, Dallas, Texas, USA; ^4^ Department of Internal Medicine, University of Texas Southwestern Medical Center, Dallas, Texas, USA; ^5^ Harold C. Simmons Comprehensive Cancer Center, University of Texas Southwestern Medical Center, Dallas, Texas, USA

**Keywords:** lung cancer, neoadjuvant chemotherapy, national cancer database

## Abstract

**Introduction:**

The current recommendation for the treatment of stage II and III NSCLC is surgery with chemotherapy. While the convention is to administer chemotherapy postoperatively (adjuvant chemotherapy), inconsistent results have been reported regarding the administration of chemotherapy preoperatively (neoadjuvant chemotherapy). Therefore, a comprehensive analysis of neoadjuvant chemotherapy use in NSCLC is needed.

**Results:**

Of the 35,134 NSCLC patients identified, 18,684 received surgery alone, 1,154 received surgery with neoadjuvant chemotherapy, and 15,296 received surgery with adjuvant chemotherapy. Race, Charlson-Deyo score, facility type, insurance type and stage of disease are associated with the use of neoadjuvant chemotherapy. In the case of stage II disease, adjuvant chemotherapy showed improved survival (median OS = 80.8 months) over neoadjuvant chemotherapy (OS = 67.0 months) and surgery alone (OS = 51.0 months). For stage III disease, adjuvant chemotherapy (OS = 49.0 months) showed improved survival over surgery alone (OS = 24.3 months), followed by neoadjuvant chemotherapy (OS = 42.0 months). After propensity score matching, adjuvant chemotherapy was found to provide a survival advantage over neoadjuvant in both stage II (HR = 0.70; *p* = 5.8e-3) and stage III (HR = 0.77; *p* = 0.011) NSCLC.

**Conclusions:**

Our analysis finds a survival advantage for neoadjuvant chemotherapy when compared to surgery alone, but no advantage compared to adjuvant chemotherapy in the treatment of resectable stage II and III NSCLC.

**Methods:**

The National Cancer Database (NCDB) was queried for all cases of stage II and III NSCLC from 2006 to 2012. These patients were stratified by stage, and the factors affecting use of neoadjuvant chemotherapy and the effects of neoadjuvant versus adjuvant chemotherapy on overall survival (OS) were investigated.

## INTRODUCTION

Lung cancer is the leading cause of cancer mortality in the United States with more than 150,000 deaths and 200,000 new diagnoses reported each year [1]. Non-Small Cell Lung Cancer (NSCLC) is a variant accounting for 85% of cases. According to the National Comprehensive Cancer Network's (NCCN), the current recommendation for initial treatment of stage II and IIIA NSCLC is adjuvant chemotherapy after complete surgical resection [2]. While studies have been unable to show an advantage of adjuvant over neoadjuvant chemotherapy, adjuvant administration remains the standard due to a larger body of evidence supporting its benefit, when compared to surgery alone [3].

A review by McElnay *et al.* found 23 randomized trials and five additional meta-analysis, conducted between 1992 and 2005, which demonstrated a survival advantage for the use of adjuvant chemotherapy for stage II and III NSCLC when compared with surgery alone [4]. More recently, the 2008 LACE meta-analysis of 2,863 patients with stage II or III NSCLC found hazards ratios for adjuvant chemotherapy of 0.83 (95% Confidence Interval [CI]: 0.73–0.95) and 0.83 (95% CI: 0.72–0.94) respectively, when compared to surgery alone [5]. One theoretical advantage of adjuvant chemotherapy is that it allows for the eradication of residual malignancy without the concurrent potential for a large primary to seed surrounding tissue. With regards to preoperative chemotherapy, many studies including the SWOG 9900 trial and ChEST trial were left incomplete after conclusive evidence supporting the benefits of adjuvant chemotherapy was published [4]. However, a 2014 meta-analysis of 15 randomized control trials, including a total of 2385 patients, reported a hazards ratio of 0.87 (95% CI: 0.78–0.96) for neoadjuvant chemotherapy when compared to surgery alone [6]. While there are fewer studies investigating the survival benefit of neoadjuvant chemotherapy, there are theoretical justifications for the use of neoadjuvant chemotherapy. These include improved tolerability, more effective dissemination through intact blood supply, and the ability to reduce tumor size before surgery [6]. One possible disadvantage of preoperative chemotherapy is the potential for leaving scattered microscopic tumor foci as the tumor shrinks thereby making complete resection more challenging [7].

Given the evidence in support of both adjuvant and neoadjuvant administration, a few studies have sought to directly compare these two modalities. A phase III trial by Felip *et al.* compared neoadjuvant and adjuvant chemotherapy to surgery alone. It found non-significant survival benefits of 0.92 (95% CI: 0.81–1.04) and 0.96 (95% CI: 0.75–1.22) for neoadjuvant and adjuvant chemotherapy respectively [8]. However, this study has been criticized for having low statistical power [9]. Additionally, 465 (75%) of the patients in the study have a clinical stage of IIA (T2b, N0) or below. A 2009 meta-analysis by Lim *et al.* compared preoperative and postoperative administration using a cohort of over 10,000 participants extracted from 32 randomized trials. This analysis reported hazards ratios of 0.81 (0.68–0.97) for preoperative and 0.80 (95% CI: 0.74–0.87) for postoperative chemotherapy. They concluded that there was no significant difference between the two approaches [9]. However, this meta-analysis included all NSCLC stages, with 27 (84%) of the studies including stage I, for which chemotherapy is not a recommended treatment option. While current research suggests there is no survival difference between neoadjuvant and adjuvant chemotherapy, this conclusion is based on a very small body of evidence. Furthermore, no studies have directly investigated this comparison in a stage-specific manner.

Our study uses a cohort obtained from the National Cancer Database to compare survival outcomes for neoadjuvant versus adjuvant chemotherapy in stage II and III NSCLC. In addition, we investigated the trend of neoadjuvant and adjuvant chemotherapy use over time as well as predictive factors associated with the receipt of one of these two approaches.

## RESULTS

### Patient cohort characteristics and treatment trend

There were 35,134 patients from 2006 to 2012 who met the study selection criteria. Of these patients, 18,684 received surgery alone, 1,154 received surgery with neoadjuvant chemotherapy, and 15,296 received surgery with adjuvant chemotherapy. The median follow-up time was 51.1 months (95% CI: 50.6–51.7). The breakdown of treatment selection by stage as well as the clinical and demographic factors for the patient cohort are shown in Table 1. For stage II patients, 13,385 (57.4%) received surgery alone, 9,387 (40.2%) received adjuvant, and 562 (2.4%) received neoadjuvant chemotherapy. For stage III patients, 5,299 (44.9%) received surgery alone, 5,909 (50.1%) received adjuvant, and 592 (5.0%) received neoadjuvant chemotherapy.

**Table 1:** Clinical and demographic characteristics of patient cohort, including patients receiving surgery alone, adjuvant chemotherapy, and neoadjuvant chemotherapy

Characteristic	Stage II (*n* = 23334)	Stage III (*n* = 11800)	*p*-value
**Age**			2.2e-06
Age (years)	66.599	66.041	
**Sex**			2.9e-05
Male	12415 (53.21%)	6557 (55.57%)	
Female	10919 (46.79%)	5243 (44.43%)	
**Race**			<2.2e-16
White	20546 (88.05%)	10270 (87.03%)	
Black	1988 (8.52%)	1078 (9.14%)	
Other	609 (2.61%)	368 (3.12%)	
**Charlson-Deyo Score**			<2.2e-16
0	11912 (51.05%)	6287 (53.28%)	
1	8193 (35.11%)	3945 (33.43%)	
2	3229 (13.84%)	1568 (13.29%)	
**Facility**			<2.2e-16
Academic	8142 (34.89%)	4473 (37.91%)	
Community	13322 (57.09%)	6351 (53.82%)	
Integrated	1633 (7.00%)	842 (7.14%)	
Other	27 (0.12%)	13 (0.11%)	
**Income**			<2.2e-16
>$63,000	6256 (26.81%)	3267 (27.69%)	
$38,000–63,000	12175 (52.18%)	6060 (51.36%)	
<$38,000	4487 (19.23%)	2235 (18.94%)	
**Insurance Type**			<2.2e-16
Private	7851 (33.65%)	4089 (34.65%)	
None	512 (2.19%)	326 (2.76%)	
Public	14719 (63.08%)	7227 (61.25%)	
**Tumor Size**			<2.2e-16
Tumor Size (mm)	40.434	52.988	
**Histology**			<2.2e-16
Adenocarcinoma	11542 (49.46%)	5883 (49.86%)	
Squamous	8150 (34.93%)	4026 (34.12%)	
Other	2457 (10.53%)	1376 (11.66%)	
**Number Chemo Agents**			0.384
Single-Agent Therapy	383 (1.64%)	232 (1.97%)	
Multi-Agent Therapy	8672 (37.16%)	5676 (48.10%)	
**Treatment**			<2.2e-16
Surgery Only	13385 (57.36%)	5299 (44.91%)	
Neoadjuvant	562 (2.41%)	592 (5.02%)	
Adjuvant	9387 (40.23%)	5909 (50.08%)	

**Figure 1:**
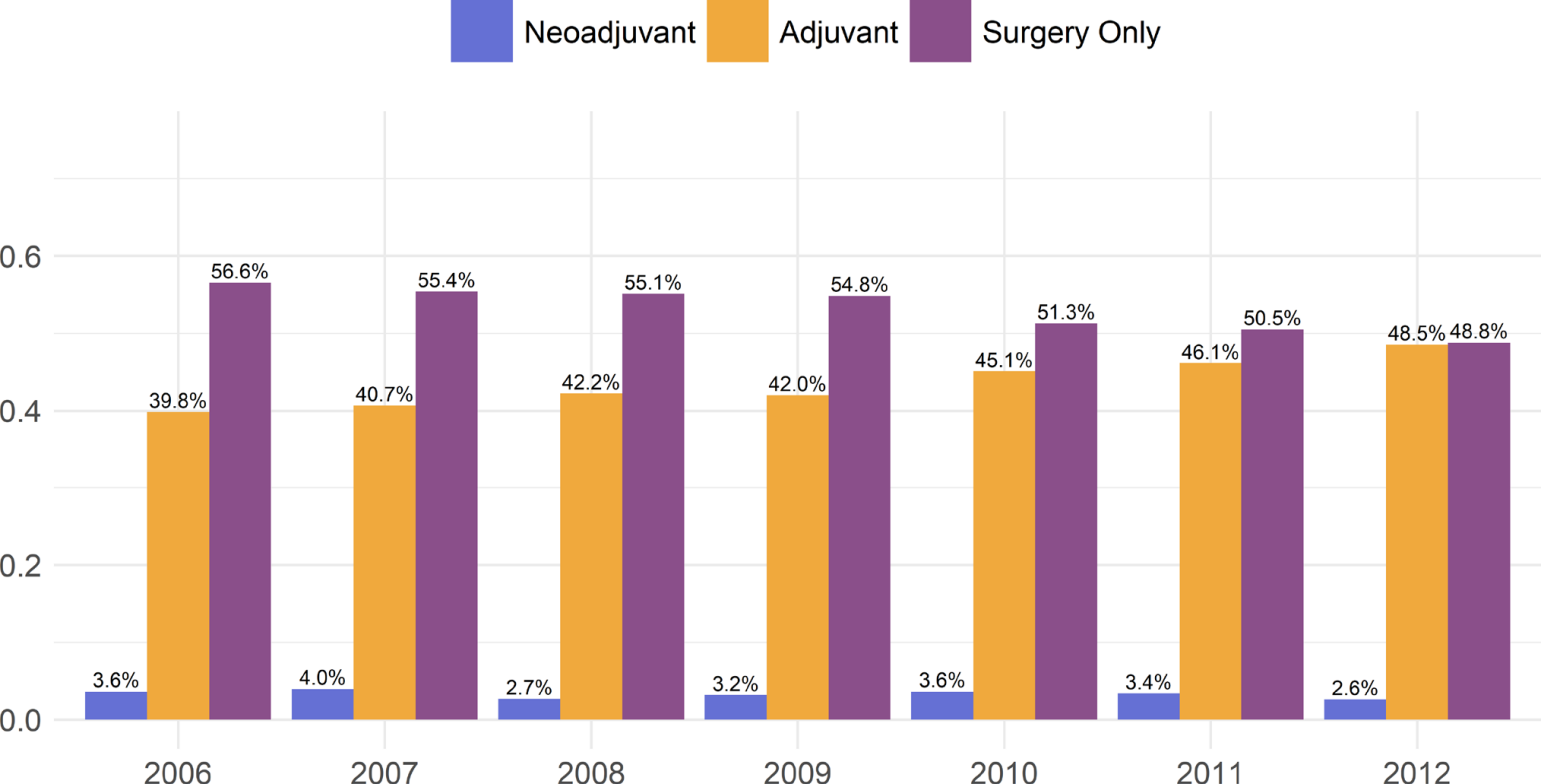
Proportion of patients receiving one of either surgery alone, neoadjuvant chemotherapy, or adjuvant chemotherapy

To investigate the treatment trend, we broke down the treatment selection by the year of diagnosis as shown in Figure 1. It clearly shows that only a small proportion of patients received neoadjuvant chemotherapy treatment, and the use of adjuvant chemotherapy steadily increased from 39.8% in 2006 to 48.5% in 2012. This clear trend is likely due to strong clinical evidence demonstrating the benefit of adjuvant chemotherapy [4]. However, the trend of neoadjuvant chemotherapy is not clear. It fluctuated between 2.6% and 4.0%. This reflects the need for a better understanding regarding the survival impact of neoadjuvant chemotherapy in lung cancer patients.

### Predictors of neoadjuvant chemotherapy use

The current clinical guidelines do not recommend neoadjuvant chemotherapy for lung cancer patients, but 3.3% of patients in our cohort received such therapy. We investigated which factors are associated with neoadjuvant chemotherapy use. Multivariable analysis of factors associated with the receipt of neoadjuvant chemotherapy versus adjuvant chemotherapy is shown in Table 2. Race of black (odds ratio [OR] = 0.67; 95% CI: 0.52–0.86; *p* = 0.002), Charlson-Deyo score of 1 (OR = 0.67; 95% CI: 0.58–0.77; *p* = 5.2e-8), treatment at community (OR = 0.52; 95% CI: 0.46–0.60; *p* < 2e-16) or integrated (OR = 0.63; 95% CI: 0.49–0.81; *p* = 4.9e-4) facilities, public insurance (OR = 0.82; 95% CI: 0.70–0.96; *p* = 0.014) or no insurance (OR = 0.53; 95% CI: 0.31–0.85; *p* = 0.014) were all significantly associated with a lower chance of receiving neoadjuvant chemotherapy. Stage III disease (OR = 1.61; 95% CI: 1.42–1.82; *p* = 8.3e-14) was associated with a higher chance of receiving neoadjuvant chemotherapy compared to stage II disease.

**Table 2:** Multivariable analysis, using the Cox proportional hazard model, of factors associated with receiving neoadjuvant chemotherapy in the National Cancer Data Base from 2006 to 2012 for stage II and III NSCLC

Characteristic	Neoadjuvant (*n* = 1154)	Adjuvant (*n* = 15296)	Odds Ratio	*p*-value
**Age (years)**				
≥64	615	8236	–	–
<64	539	7060	1.062 (0.909–1.241)	0.446
**Sex**				
Male	619	7918	–	–
Female	535	7378	0.941 (0.831–1.067)	0.344
**Race**				
White	1017	13310	–	–
Black	81	1441	0.669 (0.516–0.857)	0.002
Other	43	441	1.017 (0.714–1.408)	0.921
**Charlson-Deyo Score**				
0	734	8185	–	–
1	298	5315	0.670 (0.579–0.773)	5.2e-08
2	122	1796	0.834 (0.676–1.020)	0.083
**Facility**				
Academic	598	5507	–	–
Community	472	8548	0.524 (0.459–0.596)	<2.2e-16
Integrated	78	1111	0.634 (0.486–0.813)	4.9e-04
**Income**				
>$63,000	392	4346	–	–
$38,000-63,000	553	7962	0.872 (0.759–1.003)	0.054
<$38,000	175	2761	0.846 (0.694–1.027)	0.093
**Insurance Type**				
Private	537	6340	–	–
None	20	412	0.534 (0.313–0.852)	0.014
Public	580	8369	0.823 (0.704–0.962)	0.014
**Stage**				
II	562	9387	–	–
III	592	5909	1.606 (1.418–1.819)	8.3e-14

### Timing of chemotherapy and survival outcomes in stage II NSCLC

**Figure 2:**
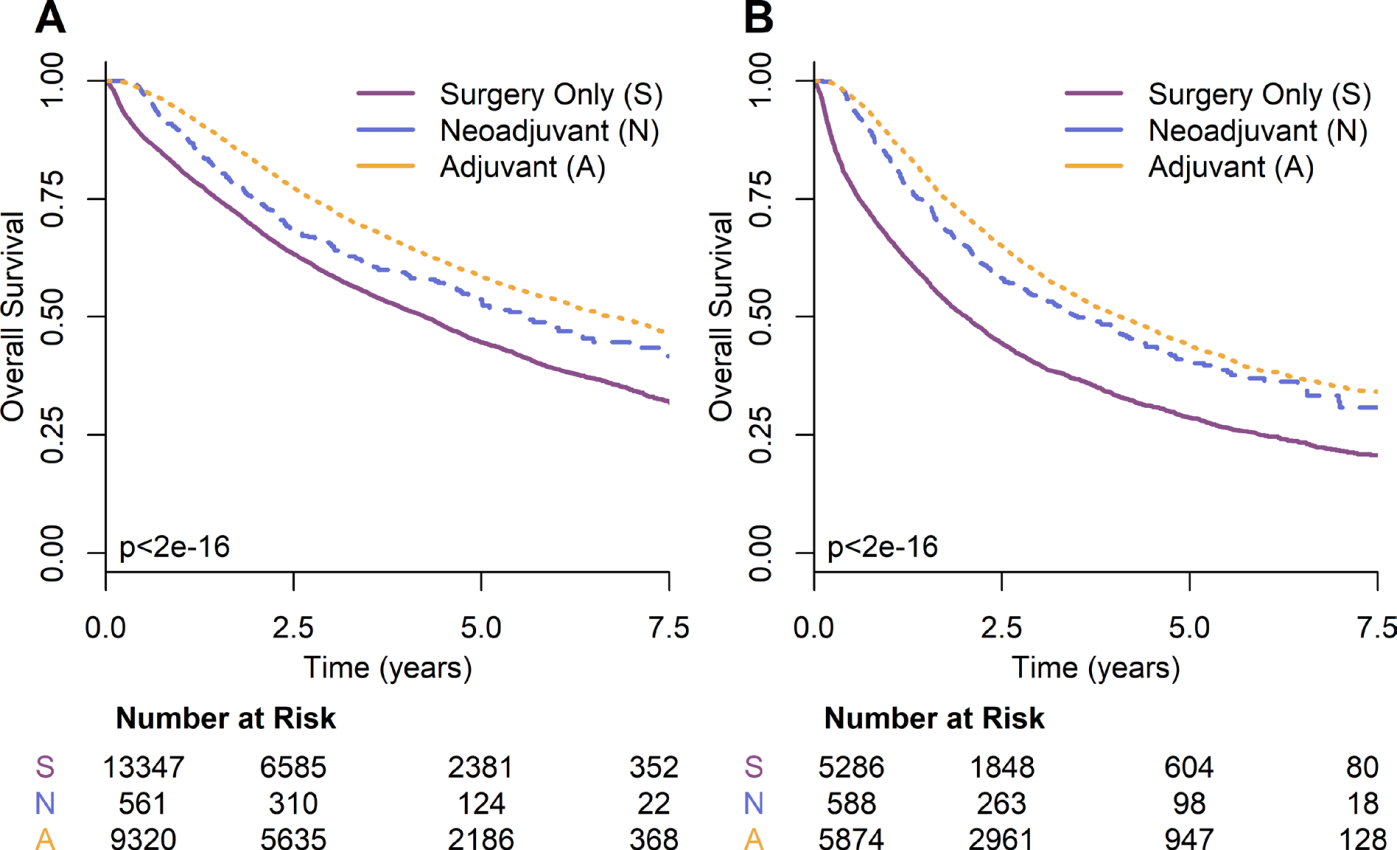
Kaplan-Meier survival curves by timing of chemotherapy for NSCLC (**A**) stage II and (**B**) stage III.

In the case of stage II NSCLC, the overall survival for surgery alone was 51.0 months (95% CI: 49.3–52.6). The greatest improvement in survival was provided by adjuvant chemotherapy (OS = 80.8; 95% CI: 77.3–85.9; *p* < 2e-16) followed by neoadjuvant chemotherapy (OS = 67.0; 95% CI: 57.9–89.4; *p* = 3.2e-5). The survival curves for stage II NSCLC are shown in Figure 2A. On multivariable analysis (Table 3), adjuvant chemotherapy was found to provide significant survival advantage over neoadjuvant (Hazards Ratio [HR] = 0.75; 95% CI: 0.65–0.88; *p* = 2.2e-4). When compared to surgery only, adjuvant and neoadjuvant chemotherapy had hazards ratios on multivariable analysis of 0.63 (95% CI: 0.60–0.66; *p* < 2e-16) and 0.82 (95% CI: 0.72–0.94; *p* = 5.3e-3) respectively.

**Table 3:** Multivariable analysis, using the Cox proportional hazard model, for predictors of overall survival in stage II and III NSCLC from 2006 to 2012 in the NCDB

Variable	Stage II		Stage III	
	HR (95% CI)	*P*-Value	HR (95% CI)	*P*-Value
**Treatment**				
Neoadjuvant	–	–	–	–
Adjuvant	0.75 (0.65–0.88)	2.2e-04	0.80 (0.70–0.91)	8.5e-04
**Age**				
≥64 years	1.26 (1.15–1.39)	7.5e-07	1.15 (1.05–1.27)	4.0e-03
**Sex**				
Male	–	–	–	–
Female	0.84 (0.78–0.90)	2.1e-06	0.79 (0.73–0.86)	1.2e-08
**Race**				
White	–	–	–	–
Black	0.92 (0.80–1.05)	0.21	0.96 (0.83–1.10)	0.57
Other	1.06 (0.84–1.32)	0.64	0.85 (0.67–1.09)	0.21
**Charlson-Deyo Score**				
0	–	–	–	–
1	1.28 (1.18–1.38)	1.4e-09	1.09 (1.00–1.19)	4.0e-02
2	1.49 (1.33–1.66)	2.5e-12	1.27 (1.13–1.42)	8.6e-05
**Facility**				
Academic	–	–	–	–
Community	1.13 (1.04–1.22)	3.0e-03	1.11 (1.02–1.20)	1.8e-02
Integrated	1.04 (0.89–1.22)	0.59	1.03 (0.88–1.20)	0.73
Other	1.05 (0.43–2.52)	0.92	1.46 (0.60–3.52)	0.40
**Income**				
>$63,000	–	–	–	–
$38,000–63,000	1.10 (1.01–1.20)	3.7e-02	1.09 (1.00–1.20)	6.1e-02
<$38,000	1.13 (1.01–1.27)	2.8e-02	1.19 (1.05–1.34)	4.9e-03
**Insurance Type**				
Private	–	–	–	–
None	1.20 (0.93–1.54)	0.15	1.03 (0.80–1.32)	0.82
Public	1.20 (1.09–1.32)	1.3e-04	1.22 (1.10–1.34)	1.0e-04
**Tumor Size**				
≥42 mm	0.90 (0.83–0.97)	5.3e-03	0.95 (0.88–1.04)	0.25
**Histology**				
Adenocarcinoma	–	–	–	–
Squamous	0.88 (0.81–0.96)	2.6e-03	0.83 (0.76–0.91)	8.6e-05
Other	1.17 (1.04–1.30)	6.8e-03	1.15 (1.03–1.30)	1.7e-02
**Number Chemo Agents**				
Single-Agent Therapy	–	–	–	–
Multi-Agent Therapy	0.88 (0.75–1.04)	0.15	0.83 (0.69–1.01)	6.1e-02

**Figure 3:**
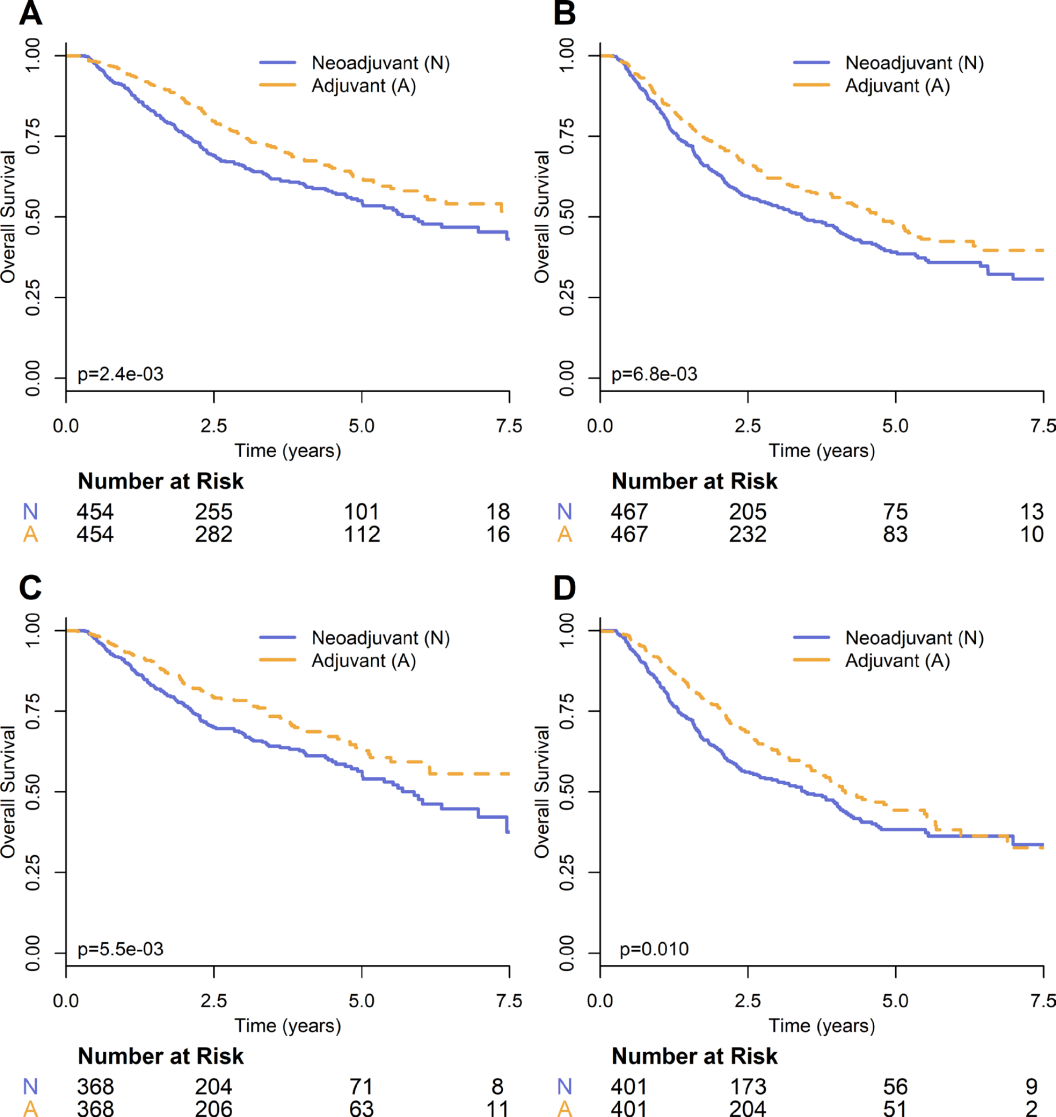
Kaplan–Meier survival curves comparing timing of chemotherapy on propensity-score matched groups for NSCLC (**A**) stage II and (**B**) stage III, and with the additional covariates of T and N stage shift for NSCLC (**C**) stage II and (**D**) stage III.

Propensity score matching on the adjuvant and neoadjuvant groups resulted in a cohort with 368 patients in each treatment group and an absolute standardized difference of 8.8e-3. Adjuvant chemotherapy was again found to provide a significant survival advantage over neoadjuvant chemotherapy (HR = 0.70; 95% CI: 0.54–0.90; *p* = 5.8e-3). Survival curves for stage II propensity score matched groups are shown in Figure 3C. Figure 3A shows survival curves for stage II propensity matched groups where the covariates of T and N stage shift are excluded.

### Timing of chemotherapy in stage III NSCLC

For stage III NSCLC the overall survival for surgery alone was 24.3 months (95% CI: 22.9–25.5). Adjuvant chemotherapy again provided the greatest improvement in overall survival (OS = 49.0; 95% CI: 46.5–52.0; *p* < 2e-16) followed by neoadjuvant chemotherapy (OS = 42.0; 95% CI: 35.9–51.4; *p* = 2.9e-14). The survival curves for stage III NSCLC are shown in Figure 2B. On multivariable analysis (Table 3), adjuvant chemotherapy provided a survival advantage over neoadjuvant (HR = 0.80; 95% CI: 0.70–0.91; *p* = 8.5e-4). When compared to surgery only, adjuvant and neoadjuvant chemotherapy had hazards ratios on multivariable analysis of 0.56 (95% CI: 0.53–0.59; *p* < 2e-16) and 0.70 (95% CI: 0.62–0.80; *p* = 4.4e-8) respectively.

Propensity score matching resulted in neoadjuvant and adjuvant groups, each with 401 patients. The absolute standardized difference between the groups was 0.017. Hazards ratio analysis on the propensity matched groups demonstrated a significant survival benefit for adjuvant chemotherapy (HR = 0.77; 95% CI: 0.63–0.94; *p* = 0.011). Survival curves for stage III propensity score matched groups are shown in Figure 3D. Figure 3B shows survival curves for stage III propensity matched groups where the covariates of T and N stage shift are excluded.

## DISCUSSION

On analysis of the National Cancer Database, our study found a significant survival advantage for the use of adjuvant over neoadjuvant chemotherapy in the treatment of stage II and III NSCLC. This finding was significant on both multivariable and propensity score analysis with the propensity matched groups demonstrating a 10.2% and 9.2% increase in three-year survival rate for stages II and III respectively. This is the first retrospective analysis to show a survival advantage for the selection of adjuvant over neoadjuvant chemotherapy.

As shown in Figure 1, the use of neoadjuvant chemotherapy ranges from 2.6% to 4.0% with a non-negligible percentage of patients receiving neoadjuvant each year. While studies have attempted to compare adjuvant and neoadjuvant chemotherapy, the results have been inconclusive, and this is reflected by the lack of a discernable trend from 2006 to 2012. Interestingly, several factors associated with poor survival, such as being treated at a community treatment center or having no insurance were associated with a greater likelihood of receiving adjuvant chemotherapy. One potential explanation for this finding is that the type of provider procured by someone with private insurance might be more likely to decline the traditional selection of adjuvant therapy based on theoretical justifications for the use of neoadjuvant. In contrast, perhaps a provider at, for example, a community-treatment center would be more likely to stick with the standard-of-care rather than considering potential benefits of a less commonly used approach.

For stage II and III NSCLC, our results indicated a significant benefit for the use of adjuvant chemotherapy over neoadjuvant with hazards ratios of 0.75 (95% CI: 0.65–0.88; *p* = 2.2e-4) and 0.80 (95% CI: 0.70–0.91; *p* = 8.5e-4) respectively. This is in comparison to the meta-analysis by Lim *et al.* which concluded that there was no difference between the approaches [9]. However, as previously noted, the study by Lim *et al.* did not restrict its analysis by stage, and included a significant number of stage I NSCLC, for which chemotherapy is not a recommended treatment. When compared to surgery alone, the hazards ratios of adjuvant chemotherapy for stage II (HR=0.63; 95% CI: 0.60–0.66; *p* < 2e-16) and III (HR = 0.56; 95% CI: 0.53–0.59; *p* < 2e-16) are much lower than that reported by the 2008 LACE meta-analysis (HR = 0.83; 95% CI: 0.73–0.95) [5]. However, the LACE meta-analysis used a patient cohort extracted from studies with inclusion periods ranging from 1994 to 2001. Advances in chemotherapy techniques may account for this difference. When neoadjuvant was compared to surgery alone, our analysis found a hazards ratio of 0.82 (95% CI: 0.72–0.94; *p* = 5.3e-3) for stage II and 0.70 (95% CI: 0.62–0.80; *p* = 4.4e-8) for stage III which is in comparison to that reported by a 2014 meta-analysis (HR = 0.87; 95% CI: 0.78–0.96). However, like the LACE study, this meta-analysis had an inclusion period ranging from 1987 to 2007. Additionally, stage I cases were included in the analysis.

Our study is limited by nature of it being a retrospective study. While we used multivariable analysis and propensity score matching to help negate the effect of confounding factors, it is possible that there were variables not captured within the database that impacted both survival and treatment selection. For example, perhaps neoadjuvant chemotherapy is more often selected in cases of borderline resectability to shrink the primary before surgery. Decisions on treatment plans depend heavily on clinical judgement and our dataset is unable to account for situations in which the provider considers the case more severe than our metrics can capture. Additionally, the NCDB only includes the number of chemotherapeutic agents administered but does not include information on the specific type of agents. Although clinical trials showed no significant survival difference among different chemotherapy regimens [10], different chemotherapy regimens may be a possible source of confounding bias that we could not capture in our analysis. It is also important to note that the NCDB does not record whether the cause of death is disease-related. Another factor that could have influenced our results is the effect of neoadjuvant chemotherapy on down-staging. It is possible that preoperative chemotherapy could have sufficiently shrunk tumor-size such that pathologic-staging information would reflect a lower-stage than the actual initial disease. We attempted to account for this factor by estimating the degree of downstaging by taking the difference between the pathologic and clinical staging, and using this as a covariate for propensity score matching. However, while this provides a reasonable estimate of downstaging, the information contained in the NCDB dataset does not allow for a more precise determination.

In conclusion, this study used a national cohort, acquired from the NCDB, to investigate survival outcomes with neoadjuvant and adjuvant chemotherapy. After analysis, we find that both adjuvant and neoadjuvant chemotherapy provide superior survival outcomes compared to surgery alone, but there is clearly no suggestion that neoadjuvant is superior to adjuvant in the treatment of resectable stage II and III NSCLC.

## MATERIALS AND METHODS

### Patient selection and variable definitions

The National Cancer Database (NCDB) collects data from more than 1500 facilities in North America, and between 1985 and 2005 was estimated to have acquired data on over 80% of new lung cancer diagnoses [11]. We queried the NCDB for all stage II and III NSCLC lung cancer patients from 2006 to 2012. We did not include patient data recorded prior to 2006 as it did not provide information on the timing of chemotherapy. Patients were excluded if the use of surgery, chemotherapy, or radiotherapy was unknown, if the timing of chemotherapy was unknown, or if both adjuvant and neoadjuvant chemotherapy was administered. Patients with positive surgical margins, or unknown margin status were also excluded. Additionally, all patients who received radiotherapy were excluded.

Staging information is provided in the NCDB consistent with the American Joint Committee on Cancer (AJCC) 6th or 7th edition depending on the year of collection. However, the 8th edition is both more applicable to current practice and has been shown to be a more reliable predictor of prognosis [12]. Therefore, the AJCC 8th edition stage [12] was used in the analysis. To account for the potential confounding factor of staging changes due to neoadjuvant chemotherapy, we defined stage shift variables by taking the difference between clinical stage and pathological stage. T stage shift was defined by taking clinical T stage and subtracting pathological T stage. Similarly, N stage shift was calculated by taking clinical N stage and subtracting pathological N stage. These stage shift variables were used in propensity score analysis to account for potential confounding.

In the multivariable and propensity score analysis, histology was included as a covariate. Histology information is coded in the NCDB according to the International Classification of Disease for Oncology, Third Edition (ICD-O-3). Cases were grouped into three categories – adenocarcinoma (8050, 8051, 8140–8147, 8230,8250–8263, 8290, 8310, 8323, 8333, 8470–8490, 8550), squamous cell carcinoma (8052, 8070, 8071, 8072, 8073, 8076, 8078, 8083, 8084, 8094), or other. In our multivariable and propensity score analysis, the covariate age was converted to a binary variable using the cohort's median age of 64 years as the cutoff.

### Statistical analysis

Chi-square test was used to test if treatment selection was different between different stages. Multivariable logistic regression was used to identify predictors of neoadjuvant chemotherapy usage.

Overall survival (OS) was the primary outcome of interest and defined as the time from diagnosis till death or last follow-up. The median follow-up time was computed using the reverse Kaplan-Meier method [13]. Survival curves were drawn using the Kaplan-Meier method and compared using the log-rank test [14]. Multivariable Cox proportional hazard model was used to evaluate the impact of prognostic predictors. To further control for confounding variables, propensity scores were calculated using multiple logistic regression including the following covariates: age, sex, race, income, type of insurance, facility type, histology, number of chemotherapy agents, and Charlson-Deyo score. The Charlson-Deyo score is a variable which captures the number and severity of comorbid conditions [15]. Based on propensity scores, different treatment groups were then matched using a nearest neighbor matching algorithm. The matched groups were considered acceptable if they had a mean absolute standardized difference of propensity scores less than 0.10 [16]. Further propensity score analysis was conducted with the additional covariates of T stage shift, and N stage shift.

All analyses were performed using R software, version 3.3.2. Packages “survival”, version 2.40-1, “MatchIt”, version 2.4-21, and “ggplot2”, version 2.2.1. The level of significance was set at 0.05, and all *p*-values were two-sided. This study was approved by the University of Texas Southwestern Institutional Review Board (IRB).
